# Post-traumatic stress disorder, food insecurity, and social capital after the 2017 coastal El Niño flooding among mothers from Piura, Peru: A mixed method study

**DOI:** 10.1371/journal.pgph.0002996

**Published:** 2024-04-18

**Authors:** Carlos Culquichicón, David Astudillo-Rueda, Roberto Niño-Garcia, Raisa N. Martinez-Rivera, Nicole Merino Tsui, Robert H. Gilman, Karen Levy, Andrés G. Lescano

**Affiliations:** 1 CI-Emerge, Center of Emerging Diseases and Climate Change, Universidad Nacional de Piura, Piura, Peru; 2 Emerge, Emerging Diseases and Climate Change Research Unit, School of Public Health and Administration, Universidad Peruana Cayetano Heredia, Lima, Peru; 3 Gangarosa Department of Environmental Health, Rollins School of Public Health, Emory University, Atlanta, Georgia, United States of America; 4 School of Medicine, Universidad Cesar Vallejo, Piura, Peru; 5 Department of International Health, Bloomberg School of Public Health, Johns Hopkins University, Baltimore, Maryland, United States of America; 6 Asociación Benéfica PRISMA, Lima, Peru; 7 Universidad Peruana Cayetano Heredia, Lima, Peru; 8 Department of Environmental and Occupational Health Sciences, School of Public Health, University of Washington, Seattle, Washington, United States of America; International Centre for Diarrhoeal Disease Research, Bangladesh (icddr,b), BANGLADESH

## Abstract

In order to understand the impacts in the post-disaster scenario of the 2017 El Niño events in the Piura region-Peru, we examined post-traumatic stress disorder (PTSD), food insecurity (FI), and social capital (SC) across three-time points in mothers in highly affected areas. In the Piura, Castilla, and Catacaos districts, we studied mothers combining mixed-method assessments at three (June-July 2017), eight and 12 months after the flooding. Each outcome was measured with the PTSD-Checklist-Civilian (PCL-C), the Household-Food-Insecurity-Access-Scale (HFIAS), the Adapted-Social-Capital-Assessment-Tool (SASCAT) surveys. In-depth interviews at the first evaluation were also conducted. At the first evaluation, 38.1% (n = 21) of 179 mothers reported PTSD; eight months and one year after the flooding, it dropped to 1.9% and virtually zero, respectively. Severe FI also declined over time, from 90.0% three months after the flooding to 31.8% eight months after, to 13.1% one year after. Conversely, high-cognitive SC was increased three months after the flooding (42.1%) and much greater levels at eight and 12 months after (86.7% and 77.7%, respectively). High levels of PTSD and severe FI three months after the flooding consistently decreased to nearly zero one-year post-disaster. High levels of high-cognitive SC may have helped mothers to recover from PTSD and FI in Piura.

## Introduction

Flooding is typically a low-mortality extreme weather event [1], but it can have substantial impacts on multiple health outcomes over the short and long term. Flooding can lead to post-traumatic stress disorders (PTSD), reaching an estimated 28% of individuals affected by large flooding events [2]. PTSD can chronically undermine well-being and mental health for up to 13 years among people exposed to floods [2–4], and it has a wide variety of manifestations, including re-experiencing trauma (69%), hyperarousal (50%), and avoidance and numbing/dysphoria (17%) [3]. In addition to mental health impacts, flooding can also limit a population’s ability to reliably access sufficient amounts of nutritious food. Food insecurity (FI) is the lack of access to food for a healthy life, and has been reported in 15% of affected inhabitants even five years after a major flood due to a hurricane [5]. Food insecurity also increases the chances of experiencing anxiety disorders, such as PTSD, [6–8] and acute events of psychological distress in 78% and 13% of individuals affected by large flooding events in low-income settings, respectively [9]. Finally, social capital (SC) is defined as “the ability of actors to secure benefits by virtue of membership in social networks and other social structures” [10]. Specifically, SC provides networks that people can access for resources or resource exchange [11], and can improve mental health outcomes and enhance connections with others, boosting adaptive responses in post-disaster scenarios. Strong social capital can diminish the chances of PTSD and related symptoms, as demonstrated in previous studies conducted after earthquakes and floodings [12, 13]. Additionally, flooding and other disasters impact significantly the health of mothers and children, increasing the risk of respiratory disease, diarrheal illness and nutritional status [14–16].

The Peruvian Ministry of Health’s guidelines for post-disaster surveillance prioritize rapid evaluations of people relocated in shelters, sanitation resources, and distribution of food resources [17]. Additionally, they recommend implementing surveillance for arboviral and other emerging diseases of high potential disease burden after extreme events, as well as mental health outcomes. However, the proposed mental health surveillance approach focuses primarily on tracking deaths due to suicide and reported cases of gender violence [17], in which the Piura region has reported 5.3–6.6 suicides per 100,000 inhabitants, and 10–17% of gender violence in 2012–17 [18, 19]. PTSD is not part of post-disaster assessments in spite of recommendations from a study after the 2007 Ica earthquake [20]. Similarly, changes on dietary patterns among mothers, and infants were observed after the 1998 El Niño event in the northern coast and the 2011 La Niña-related flooding in the Peruvian Amazon [21, 22], and food donations are part of humanitarian relief in disasters. However, food security evaluations are not part of post-disaster surveillance guidelines, which can be critical in vulnerable regions such as Piura where 10–20% of children already presented stunting in the last decade [19, 23]. Therefore, the impact of post-disaster response efforts could be enhanced by including screening and monitoring for major mental health disorders, as well as assessing food insecurity and the social capital of affected populations [17, 24].

The 2017 coastal El Niño event dramatically affected the northern Pacific coast of Peru, causing a critical period of high rainfall along with landslides and extensive floods [25]. On March 27^th^, several levees holding back the Piura river broke, and the river flooded urban as well as farming areas in the cities of Piura, Castilla and Catacaos and their outskirts [25]. Approximately 335,000 houses were severely damaged and 92,896 inhabitants across the Piura region were affected by the rainfall and flooding [25]. Over 63,000 people were affected in the Piura province, and three months after the disaster, 37,100 people in Catacaos and 14,400 in Castilla had still not fully recovered [25].

In late 2015, our group had started a small cohort study in Piura to evaluate the potential impact of the expected 2015–2016 El Niño. Therefore, the extreme 2017 flooding event allowed us to estimate the frequency of PTSD and food insecurity among affected mothers in the Piura region, to document its evolution over time, and to examine if and how social capital mitigated the impacts of flooding.

## Methods

### Study design

We conducted a mixed-methods assessment of PTSD, food insecurity, and social capital among mothers enrolled in a cohort study established prior to the El Niño event, using both structured surveys and in-depth interviews from June 3^rd^, 2017 to April 14^th^, 2018. Our study had a first evaluation on June-July 2017, approximately three months after the flooding, and two more evaluations conducted on November-December 2017 and February-April 2018.

### Population and sample

Piura is a northern coastal region in Peru inhabited by approximately 1.8 million individuals, 450,000 of whom live in the city of Piura [26]. It is estimated that Piura has experienced at least 120 El Niño events over the past five centuries [27]. The most severe El Niño events occurred in 1891, 1925, 1982/83, 1997/98, and 2017. The most recent one, in February-April 2017, had a major impact on human health on the northern coast of Peru [27].

In February 2017, the El Niño Multisectoral Committee identified oceanic Kelvin heat waves moving south from equatorial seas to the coast of northern Peru. These abnormal oceanic heat waves increased the sea surface temperatures by 1.5°C on average across Peru’s central and northern coast [28]. Due to its coastal origin and the extension of the heat waves, this climatic condition was termed a “coastal” El Niño event [28], as opposed to the traditional El Niño events that typically originate in the 2–3 Central Western Pacific region. Between February and March of 2017, the oceanic temperature on the Peruvian coast gradually increased and peaked on March 27^th^, 2017. During this period, rainfall in the Piura province was consistently high with rates of 40–81 mm/hour, compared to its historical 4 mm/hour average, and in the upstream Andean provinces monthly precipitation rates were 137 mm/hour versus historical averages of 15 mm/hour [29]. The Piura river volume progressively increased and eventually reached 3,468 m^3^/sec, surpassing the 3,100 m^3^/sec security threshold for extreme-events [30]. The natural levees broke in three points, and ultimately flooded the districts of Piura, Castilla and Catacaos. The more rural and vulnerable homes in Catacaos suffered greater damage, and immediately after the flood most of the affected population was relocated in shelters arranged by non-governmental, and governmental organizations including the Peruvian army humanitarian response team. **S1 Fig** shows light-blue shaded areas affected by the flooding, and red shaded areas of basins without-mouth which the nearest to the river were affected by the flooding, and the farthest by high-constant rainfall during El Niño season.

The assessment presented here was conducted within a “parent” pediatric cohort study that assessed infant nutritional status before, during, and after El Niño events that started due to the expectation of a potential El Niño event in late 2015. Mothers in the parent cohort study were recruited when giving birth in public hospitals of Piura city and therefore are representative of most of the population in the Piura, Castilla, and Catacaos districts of the city where 93% of births take place in such public health facilities. The parent cohort had already enrolled two groups of infant-mother dyads in 2015 and 2016, and the enrollment of the 2017 group was underway in February-March before the flooding and was eventually included in the study. Therefore, in June-July 2017 we conducted a first evaluation by assessing PTSD, food security, and social capital among the mothers of the recruited infants. We prioritized mothers residing in the most affected neighborhoods of the Piura, Castilla and Catacaos districts, according to the assessment of the Piura Emergency Operations Center [29, 31]. Two additional evaluations were then conducted in November-December 2017 and February-April 2018.

### Data collection

We contacted all eligible mothers enrolled in the parent cohort study over the phone to schedule in person visits before any evaluation, and applied a structured survey that evaluated PTSD, food insecurity, and social capital. In each time point, we tried to direct the assessments’ questions to ask only about outcomes directly related to the flooding event. Additionally, in the second and third assessments, we asked the mother about both prevalent and new, incident outcomes. For example, in the July 2017 assessment, the interviewers asked specifically the impact of the El Niño event as follows: "Did you **ever** feel the following issues **during the period of high rainfall, river floods, and the following weeks** (…)”. In the last two evaluations, the interviewers evaluated the outcomes derived from the El Niño events as follows: “**Since the last visit**, have you experienced any of the following issues (…) **due to the period of high-rainfalls, river floods, and the following weeks**?”.

In addition, in the first post-disaster evaluation we conducted semi-structured in-depth interviews only on individuals with PTSD or severe food insecurity to understand potential reasons and individual factors triggering and contributing to these conditions. In-depth interviews further explored on the mother’s PTSD and food insecurity responses with highest severity/risk scores as time allowed, and were conducted by locally-trained professionals familiar with regional norms and local idioms and recorded after asking the permission of the participant.

### Instrument and scales

PTSD was evaluated using the PTSD Checklist-Civilian (PCL-C), which specifically measures the reexperiencing (B), avoidance and numbing/dysphoria (C), and hyperarousal (D) domains of PTSD. This test is based on the Diagnostic and Statistical Manual of Mental Disorders—4 (DSM-4) and has been validated in Latino populations in the United States [32], and in a research study in Peru [20]. It uses 17 Likert scale-formatted questions of 1–5 points to diagnose PTSD (>50 points) following the National Center for PTSD’s rubric [33]. Symptomatic individuals are those with ≥3 points (moderate to extreme) for at least one B item, three C items, and two D items.

Food insecurity was evaluated using eight of the nine items of the Household Food Insecurity Access Scale (HFIAS) [34], but excluded item five because it was very similar to item six and participants found it confusing during the pilot study. Items were worded to address specifically the flooding context, and responses follow a 0–3 Likert scale: 0:never, 1:rarely, 2:sometimes and 3:frequently. Food insecurity was determined following the FANTA-III’s rubric [34]: low if participants responded 2–3 on item one, 1–3 on item two, or 1 on items three or four, moderate participants responded 2–3 on items three or four, or 1–2 on items five or six, and severe if participants responded three on items five or six, or 1–3 on items seven or eight. The full scale has been validated in Spanish for Latino populations in the United States [34].

Social capital reflects a population’s connectedness and the quantity and quality of its social relationships using two components. Structural social capital describes the community’s capability to build relationships with local and external organizations, and cognitive social capital represents the support, reciprocity, solidarity, and trust of individuals to the social relationships established [35]. We evaluated both capital components with the 41-item Adapted Social Capital Assessment Tool (SASCAT), previously validated in Latino populations in the US and used in Peruvian studies [20, 36]. High cognitive social capital was defined by a positive response to three or more out of the four cognitive component items that assess community perceptions of trust, sense of belonging and internal harmony [36, 37]. Structural social capital was characterized through seven components and sub-components including: group membership, supporting groups, support from groups and individuals, and activities in benefit of the community.

### Data analyses

We evaluated the reliability of the PCL-C, HFIAS and SASCAT scales applied by estimating their Cronbach’s alpha. Changes in the frequency of PTSD, food security and high cognitive social capital between post-disaster evaluations were evaluated using Chi^2^ tests with bootstrapping. Similarly, changes in global PCL-C and HFIAS scores and SASCAT individual item responses were evaluated with Student’s T-tests, and ANOVA with Bonferroni correction for multiple comparisons. All analyses was performed using Stata 15.1 ED using a 5% significance level, and the analysis code is openly available in git-hub [38].

Intercoder reliability was first conducted between three trained team members, and an anthropologist. Tape recordings of in-depth interviews were transcribed verbatim by the interviewers. Thematic codes were assigned to sections in the interview transcripts, and the main themes and sub-themes were identified by analyzing the frequency and content of these codes, linked to the domains of the validated scales applied in the questionnaires [39]. We used the Atlas.ti software [40] for coding and qualitative analysis.

### Research ethics

This research project was conducted as part of El Niño cohort study which was approved by the institutional review board of the Asociación Benéfica PRISMA (CE1209.17). All participants provided written consent prior to any data collection and tape recording. All participant names described in this manuscript were made up by the authors in order to protect participants’ true identities. Participants were not incentivized for participation. Additional information regarding the ethical, cultural, and scientific considerations specific to inclusivity in global research is included in the Supporting Information (S1 Checklist).

## Results

At the time of our study, there were 350 mothers readily available from the parent cohort study (cohort groups 2015, 2016, and 2017). Not all enrolled mothers were assessed because of geographical inaccessibility at the first post-disaster evaluation and relocation due to the flooding (n = 51), withdrew to parent cohort study follow-ups (n = 43), and loss to follow-up (n = 77). Thus, a total of 179 mothers were included in this study (**S2 Fig**).

Mothers with one, two, and three visits were 95, 64, and 1, respectively (N total mothers visited = 160), and a total of 226 assessments were conducted adding the three post-disaster evaluations. On average, mothers in the study were 27.2±7.2 years old (mean ± standard deviation), had 2.2±1.4 children, and 64% completed high school or technical education (**Table 1**). Across the three evaluations, mother’s houses were primarily built with bricks, galvanized roof panels, with interrupted in-house water connections, and without sewage connection (**Table 1**). The socio-demographic characteristics by cohort groups are presented in **S1 Table**.

**Table 1:** Socioeconomic conditions of interviewed mothers affected by the 2017 El Niño flooding by follow-ups in the Piura region, Peru, 2017–2018.

Characteristics	N = 226	%	Follow-ups					
			June-July 2017 (N = 21)		November-December 2017 (N = 111)		February-April 2018 (N = 94)	
			n	%	n	%	N	%
Age (years)	27.2±7.2		28.2±7.1		27.5±6.9		26.7±6.6	
Siblings	2.2±1.3		2.7±1.3		2.2±1.3		2.1±1.1	
Job								
Employee	31	13.7	2	9.5	17	15.3	12	12.8
Student	17	7.5	1	4.8	8	7.2	8	8.5
Home maker	178	78.8	18	85.7	86	77.5	74	78.7
Residence location								
Piura	107	47.4	2	9.5	51	46.0	54	57.5
Castilla	48	21.2	3	14.3	26	23.4	19	20.2
Catacaos	71	31.4	16	76.2	34	30.6	21	22.3
Education								
Elementary	13	5.9	2	10.0	6	5.6	5	5.4
High school	70	31.7	12	60.0	30	27.8	28	30.1
Technical	96	43.4	5	25.0	51	47.2	40	43.0
University	42	19.0	1	5.0	21	19.4	20	21.5
Household-walls construction material								
Tapial (rocks with mud)	6	2.7	2	9.5	3	2.7	1	1.1
Quincha (cane with mud)	26	11.6	6	28.6	9	8.2	11	11.8
Mud bricks	22	9.8	1	4.8	13	11.8	8	8.6
Hardwood plywood	46	20.5	2	9.5	22	20.0	22	23.7
Red concrete rick	124	55.4	10	47.6	63	57.3	51	54.8
Household-roof construction material								
Galvanized roof panels	159	71.0	18	85.7	73	66.4	68	73.1
Fiber cement	33	14.7	3	14.3	18	16.4	12	12.9
Brick	32	14.3	0	0.0	19	17.3	13	14.0
Water source								
Public joint connection	12	5.4	0	0.0	9	8.3	3	3.3
Tank truck	3	1.4	0	0.0	2	1.8	1	1.1
Neighbor	16	7.2	1	4.8	7	6.4	8	8.7
Interrupted in-house connection (<24 hours/day)	133	59.9	13	61.9	64	58.7	56	60.9
Continuous in-house connection (24 hour/day)	53	23.9	6	28.6	24	22.0	23	25.0
Front house connection	5	2.3	1	4.8	3	2.8	1	1.1
Fuel for cooking								
Coal	18	8.0	1	4.8	8	7.3	9	9.7
Wood	38	17.0	4	19.1	19	17.3	15	16.1
Gas	168	75.0	16	76.2	83	75.5	69	74.2
Color television								
None	33	14.7	4	19.1	15	13.6	14	15.1
One	148	66.1	15	71.4	69	62.7	64	68.8
Two or more	43	19.2	2	9.5	26	23.6	15	16.1
Toilet facilities								
Land	20	8.9	1	4.8	11	10.0	8	8.6
Silo	41	18.3	3	14.3	19	17.3	19	20.4
Cesspool	7	3.1	2	9.5	3	2.7	2	2.2
Latrine	11	4.9	1	4.8	6	5.5	4	4.3
Drain	145	64.7	14	66.7	71	64.6	60	64.5
Garbage disposal								
Land	14	6.3	2	10.0	7	6.4	5	5.4
Burning	20	9.0	2	10.0	11	10.1	7	7.5
Containers	13	5.9	0	0.0	9	8.3	4	4.3
Garbage truck	175	78.8	16	80.0	82	75.2	77	82.8
Animal breeding								
No	71	31.7	3	14.3	36	32.7	32	34.4
Yes	153	68.3	18.0	85.7	74	67.3	61	65.6

### Post-traumatic stress disorder

**Fig 1:**
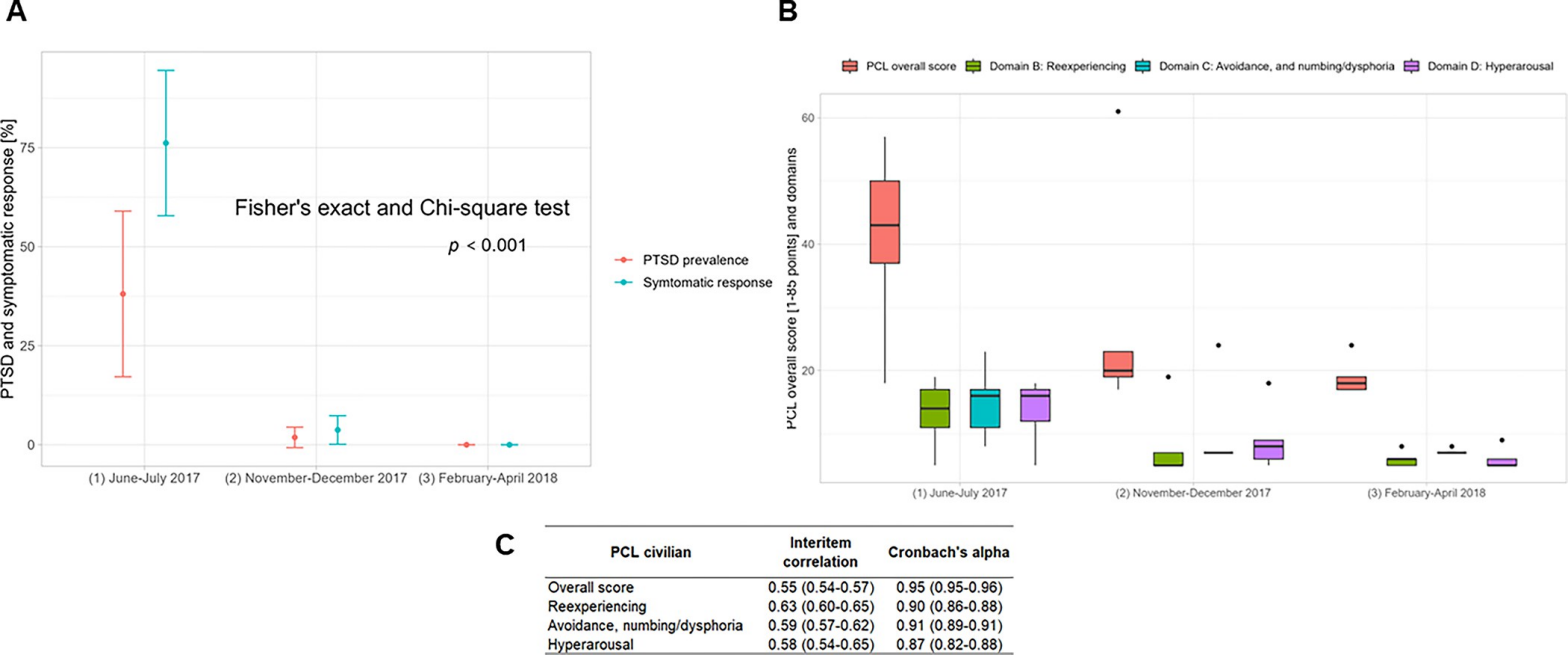
Impact of the 2017 El Niño flooding on post-traumatic stress disorder (PTSD) of mothers in the Piura region, Peru, 2017–2018. **(A)** Prevalence of PTSD and related-symptoms. **(B)** PCL-civilian overall and domain scores. **(C)** PCL-civilian and domain inter-item correlations and Cronbach’s alpha.

Three months after the floods (June-July 2017), 38.1% (n = 8) of mothers reported PTSD, and 76.2% (n = 16) reported at least one potential symptom of PTSD. Eight months after the crisis, only 1.9% of mothers reported PTSD and 3.7% reported PTSD-related symptoms. Approximately one year after the flood, PTSD and symptomatic response levels had diminished to close to zero. The prevalence of PTSD (Fisher’s exact probability <0.001) and symptomatic response (χ^2^, P <0.001) differed across follow-ups (**Fig 1A**). The internal consistency of the PCL-C scale was 0.95 with an inter-item correlation of 0.55 (**Fig 1C**).

The mean difference of the reexperiencing domain scores showed a decrease from June to November 2017 (-6.9, 95% CI: -8.1, -5.7; P< 0.001) and marginally decreased in November 2017 and February 2018 evaluations (-0.8, 95% CI: -1.4, 0.1; P = 0.93). Consistently, the mean difference of avoidance and numbing/dysphoria domain scores decreased from June to November 2017 (-7.5, 95% CI: -8.8, -6.2; P< 0.001); and marginally decreased for November 2017 and February 2018 evaluations (-0.8, 95% CI: -1.6, -0.1; P = 0.03). Finally, the mean difference of the hyperarousal domain scores decreased from June to November 2017 (-6.3, 95% CI: -7.5, -5.1; P< 0.001); and decreased for the November 2017 and February 2018 evaluations (-2.1, 95% CI: -2.8, -1.4; P< 0.001) (**Fig 1B**).

The in-depth interviews confirmed that during the floods and the weeks that followed, participants experienced a wide range of numbing/dysphoria symptoms, such as detachment from their families, reduction of the affection given, and a loss of interest in their relatives. For example, one mother stated:

“*I did not give much affection to my child and my husband*, *he noticed that I was sad*, *I was very disappointed*.*” Maria*, *16 years old (June 2017*, *Piura)*.

Participants also felt anxiety about the future, fearing that another flood could affect their families and houses. Another mother reported:

“*When we got home*, *we were very afraid that the same would happen again” Ofelia*, *27 years old (July 2017*, *Catacaos)*.

Participants commonly experienced hyperarousal symptoms, including sleeping disorders and insomnia, as well as acute events of distress and crying. For example:

“*I slept very little*, *I wake up at midnight*, *then I was not able to fall sleep again” Matilda*, *27 years old (July 2017*, *Castilla)*.

*“Suddenly, I would wake up at night and cry because of all the circumstances I lived through in the disaster” Clotilda (July 2017, Castilla)*.

One year after the disaster, mothers reported avoiding evocation of El Niño memories, consistently with extended presentation of PTSD-related symptoms. And some of them claimed to do chores or other activities to avoid such unpleasant memories, as described below:

“*We don’t want to think about what happened” Jimena*, *37 years old (February 2018*, *Catacaos)*.

“*I don’t like remembering or thinking about the things that I saw” Rosa*, *16 years old (March 2018*, *Castilla)*.

“*I try to use my time in different activities*, *so I do not have to remember or think about what happened” Adriana*, *27 years old (March 2018*, *Piura)*.

### Food insecurity

**Fig 2:**
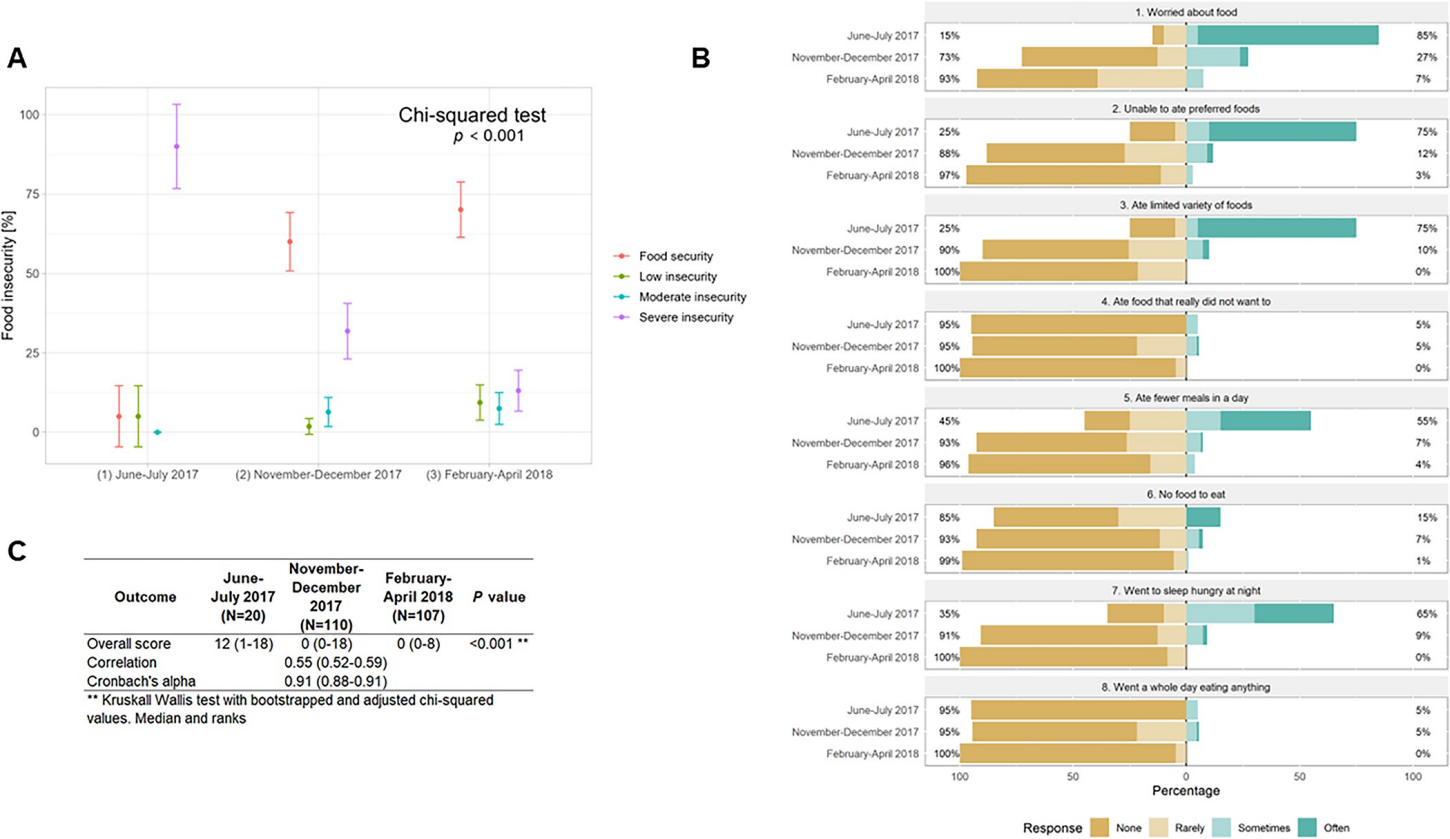
Food insecurity among mothers affected by the 2017 coastal El Niño flooding in the Piura region, Peru, 2017–2018. **(A)** Food insecurity prevalence. **(B)** HFIAS item scores. **(C)** HFIAS inter-item correlation and Cronbach’s alpha.

Three months after the flooding severe food insecurity was still remarkably high (90.0%, n = 18), but declined consistently to 31.8% (n = 35) and then to 13.1% (n = 14) over the last two assessments (**Fig 2A**). Conversely, food security was low (5.0%, n = 1) three months after the floods, albeit with a small sample size, then increased substantially eight months after the floods (to 60.0%, n = 66) and increased slightly more approximately one year after the floods (70.0%, n = 75) All the levels of food insecurity differed across follow-ups (χ^2^, P < 0.001) (**Fig 2A**).

Three months post-disaster, participants reported substantial food security concerns and challenges: they worried about having food (85.0%, n = 17), ate limited variety of food (75.0%, n = 15), were unable to eat their preferred food (75.0%, n = 15), slept hungry at night (65.0%, n = 13), and ate fewer meals in a day that normally (55.0%, n = 11) (**Fig 2B**). In the first post-disaster evaluation, all these issues were consistently less frequent, and further decrease in the second evaluation.

In-depth interviews documented that during the first month post-disaster in the Piura and Castilla districts, most mothers felt that different food items were scarce in local markets and grocery stores. In addition, food prices had increased and merchants even restricted the maximum sales per family. The following excerpts summarize some of these concerns:

“*Three days after the flood we went to the local supermarket to get some food and save for the upcoming weeks*. *When we got there*, *there was nothing*, *they were completely empty*. *It was overwhelming” Rosa*, *36 years old (July 2017*, *Castilla)*.

“*In April*, *all the food prices at grocery stores were more expensive than ever and were insanely unaffordable*. *Most of the time*, *there was no rice*, *tuna*, *noodles*, *and other essential food supplies” Luisa*, *31 years old (June 2017*, *Piura)*.

In Catacaos, the extent of the affected areas and the scarcity of supplies forced some donors and agencies to provide only modest amounts of food. Some families reported walking long distances to receive food donations in other neighborhoods. Such harsh conditions limited the parents from providing enough food to feed their families. Going to sleep hungry was a commonly reported situation and in extreme and desperate cases, mothers reported eating spoiled food when they had nothing else to feed themselves:

“*My husband and I slept starving three or four days*. *We got only one meal for us over 4 days” Andrea*, *22 years old (June 2017*, *Catacaos)*.

“*I thought I won’t get any food*, *but when I went to Montesullon (a 20 minutes trip in motocart)*, *nearby Catacaos*, *people gave us less than 500 grams of noodles” Fatima*, *39 years old (June 2017*, *Catacaos)*.

“*We ate spoiled fruit for three days because we were starving*. *We gave our food to our three kids” Gianela*, *28 years old (June 2017*, *Catacaos)*.

### Social capital

In the first three months after the floods, 72.2% (n = 15) of participants received support, either emotional (44.4%, n = 9), material (44.4%, n = 9), or as knowledge (50.0%, n = 11) from at least one organization they were involved with (**Table 2**). The prevalence of supportive groups differed across follow-ups (χ^2^, P < 0.001) In addition, 94.5% (n = 20) of participants received support from individuals such as relatives, neighbors, community religious or political leaders, or governmental authorities and NGO officers. Support from groups and individuals consistently diminished during the year after the floods. The prevalence of high cognitive social capital increased from 42.1% (n = 9) three months after the floods, to 86.7% (n = 93) eight months after the floods and remained high (77.7%, n = 82) one year after the flooding (**Table 2**). The levels of cognitive social capital differed across follow-ups (χ^2^, P < 0.001), suggesting how community members internally leaned on each other more and more during challenging times.

**Table 2:** Cognitive and structural social capital among mothers affected by the 2017 El Niño flooding in the Piura region, Peru, 2017–2018.

Parameter	June-July 2017 (N = 21)	November-December 2017 (N = 107)	February-April 2018 (N = 106)	*P* value*
Group membership				<0.001
None	44.4%	74.1%	88.5%	
One	22.2%	25.9%	11.5%	
Two or more	33.3%	0.0%	0.0%	
Supporting groups				<0.001
None	27.8%	80.4%	98.1%	
At least one	72.2%	19.6%	1.9%	
Support from groups				<0.001
Emotional	44.4%	8.3%	1.9%	
Supplies	44.4%	13.0%	0.0%	
Knowledge	50.0%	3.7%	0.0%	
Support from individuals				<0.001
None	5.6%	77.4%	91.3%	
One	27.8%	17.0%	8.8%	
Two or more	66.7%	5.7%	0.0%	
Activities in benefit of the community				<0.001
None	21.1%	65.7%	90.4%	
One	57.9%	5.6%	0.0%	
Two	21.1%	28.7%	9.6%	
Cognitive social capital				<0.001
Low	57.9%	13.3%	22.3%	
High	42.1%	86.7%	77.7%	

* Chi-squared test

Enrolled mothers were members of multiple government-funded and community-led social programs that provide food or meals to disadvantaged infants and adolescents (22.2%, n = 5), including the “Glass of Milk” and the “Mothers’ club” groups among others (**Table 3**). Only 11.1% (n = 2) of mothers participated in religious groups that primarily provided emotional support (38.9%, n = 8). No mothers participated in health or water committees but many reported receiving educational support from such groups (38.9%, n = 8) (**Table 3**). From June to July 2017, mothers were primarily supported by their relatives (78.9%, n = 17), religious representatives (42.1%, n = 9), and neighbors or friends from the neighborhood (31.6% [n = 7] each) (**Table 4**).

**Table 3:** Support from emotional, material, and learning groups during the 2017 El Niño flooding in the Piura region, Peru, June-July 2017.

Group (n = 21)	Membership (%)	Emotional (%)	Supplies (%)	Learning (%)
Community group	.	.	5.6	.
Food supply	22.2	.	5.6	.
Politic	5.6	.	.	.
Religious	11.1	38.9	22.2	.
Social/sports	.	.	.	5.6
Health/water/electricity	.	.	5.6	38.9
Elementary school	16.7	5.6	.	5.6
Security	.	.	5.6	.
No membership/no support	44.4	55.6	55.6	50.0

**Table 4:** Support from individuals during the 2017 El Niño flooding in the Piura region, Peru, 2017–2018.

Individuals	June-July 2017 (N = 21)	November-December 2017 (N = 107)	February-April 2018 (N = 106)	P value*
Relatives	78.9%	16.7%	7.7%	<0.001
Neighbors	31.6%	4.6%	.	<0.001
Friends out of the neighborhood	31.6%	3.7%	.	<0.001
Community representatives	10.5%	2.8%	.	<0.001
Religious	42.1%	1.9%	.	<0.001
Politicians	.	1.9%	.	<0.001
Government	10.5%	0.9%	.	<0.001
City hall	15.8%	2.8%	.	<0.001
NGOs	22.2%	3.8%	1.0%	<0.001

* Chi-squared test

The populations’ trust in their own communities was severely affected by the disaster. Most of the mothers expressed safety concerns in the areas impacted by the flooding, such as frequently seeing thieves trying to ransack houses. In response, parents started night shifts to protect the belongings that remained in their damaged houses, while the rest of the family stayed in shelters:

“*I was overwhelmed because people came to steal our stuff*, *my husband decided to spend every night in the house and return to the shelter in the morning” Maria*, *28 years old (July 2017*, *Piura)*.

Community members also reported distrust of governmental authorities due to their perception of lack of governmental support during the floods. However, participants stood up to support their neighbors to ensure community welfare and safety:

“*The government has not helped us at all*. *Only the volunteers who came from outside have given us food” Tania*, *20 years old (November 2017*, *Catacaos)*.

“*The mayor said that nothing had happened here*. *People were outraged and upset*. *We organized a strike to protest against this injustice” Helena*, *22 years old (February 2018*, *Catacaos)*.

Despite the devastating experience, there was substantial collaboration between residents of affected communities. Community leaders arranged groups to carry out various activities such as cooking and feeding people and restoring affected assets, as reported below:

“*Nowadays*, *we are cooking in a “joint/shared pot” to feed most of the people in the shelter” Helena*, *22 years old (February 2018*, *Catacaos)*.

“*Quite a few neighbors joined to clean-up the streets because Easter was coming” Sandra*, *17 years old (March 2018*, *Catacaos)*.

## Discussion

Even by low-mortality disasters such as floods, landslides, low-intensity earthquakes, and volcanic activity can importantly impact the mental health of populations, among other outcomes. In the Piura region, major flooding and rainfall due to the 2017 coastal El Niño immediately generated a high prevalence of post-traumatic stress disorder (PTSD) and severe food insecurity among mothers. One year after the crisis, it seems that high-cognitive social capital helped them to recover.

### Post-traumatic stress disorder

During the immediate months after the floods (from June to July 2017), the prevalence of PTSD and related-symptoms were remarkably high (38.1%, and 76.2%, respectively). This is consistent with previous work done in Peru by the post-disaster surveillance system deployed after low-mortality disasters, which shows that outpatient consultations for psychological disorders range from 18.4 to 71.5 by 100 person-years in coastal, Andean, and Amazon areas [1].

In the last two evaluations, participants reported declining PTSD and related-symptoms over time, reaching virtually zero one year after the crisis. This rapid mental health recovery may be due to previous exposure to El Niño events, as most mothers were already alive during the 1998 El Niño event [21] and one even reported experiencing the 1983 El Niño. In contrast, victims of non-recurrent events such as the 2007 Ica earthquake in southern Peru had no previous exposure to severe earthquakes, and four years after the event, 16% still reported chronic PTSD and 52% reported high-cognitive social capital [20].

In this context, we hypothesize that given the repetitive exposure to El Niño events, the inhabitants from Piura may have developed resilience patterns to face, adapt, and deal with traumas [41], and can reduce PTSD in post-disaster scenarios [42–44]. Social support allows affected individuals to use their individual and community relationships to improve their health and well-being and potentially return to pre-disaster levels [45–47]. This is critically important for socially-vulnerable populations.

### Food insecurity

Three months after the El Niño event (from June to July 2017), severe food insecurity remained remarkably high (90%). The challenging post-disaster scenario increased the affected families’ job insecurity and ultimately impacted people’s food insecurity, especially in the immediate weeks after the flooding. In our study location, many families experienced such meaningful housing losses driving to face major financial challenges to secure their family’s needs, which probably impacted directly their adequate food access and security.

In addition to the lack of families’ income for food purchases, El Niño events generated a lack of food source specially three following months of post-disaster. El Niño events severely affected 14,800 hectares and destroyed 8,700 hectares of crops in the Piura region [25]. Moreover, 29,100 kilometers (km) of urban and interdistrict roads were affected, and 320 km destroyed, such that transit for supplies and access for humanitarian response were severely diminished across the Piura region [48]. During the first month of immediate emergency, multiple agencies, religious organizations and donors made joint efforts to bring humanitarian response [25].

Humanitarian assistance probably helped families to sustain the critical post-disaster period while they seek permanent resources. Six months after the flooding, only 26% of shelter residents could buy food supplies on their own [49]. The prevalence of food insecurity eventually dropped to 13% one year post-disaster probably reflecting the presence of vulnerable groups still living in shelters and with lack of steady employment [50]. In addition, there may be people already living in food insecurity before the flooding. Overall, the displacement due to severe household damage, the inability of accessing food, the limited outreach of humanitarian assistance, and the chronic poverty of the affected communities in Piura converged to worsen their food insecurity.

Food insecurity is not only a frequent outcome in post-disaster scenarios, but it also acts as a stress factor for mental health conditions [51]. Food insecurity has been associated with developing anxiety disorders [51], and this may especially be true in traumatic events such as El Niño. Hence, prompt humanitarian response, access to food and an expedited return to pre-disaster conditions would likely enhance food security and ultimately contribute to reduce PTSD and related symptoms among disaster-affected populations such as those suffering the 2017 El Niño flooding.

### Social capital

Three months after the El Niño events, 42% of mothers reported high-cognitive social capital and it progressively increased to 87% at eight months, and remained at 78% at 12 months after the disaster, together with a sharp decline in PTSD rates. Similar increases in high-cognitive social capital and drops in PTSD prevalence have been reported also after high-mortality disasters in Peru and elsewhere [20, 52]. Such rapid mental health recovery may be influenced by community resilience patterns nurtured by strong social capital, especially in populations with repetitive exposures such as El Niño events.

We observed that inhabitants from affected communities in Piura quickly developed a collective structural social capital that allowed them to face the acute post-disaster scenario, probably based on components such as mutual trust, reciprocity, and support between individuals. Inhabitants affected progressively switched to building high cognitive social capital among themselves, which probably helped them to individually overcome such stressful situations. Cognitive social capital has been shown to reduce PTSD one year post-disaster [52], and to reduce the prevalence of chronic PTSD by 46% four years post-disaster [20]. Therefore, both at the collective and individual level, social capital appeared to substantially enhance the inhabitant’s responses to overcome the mental health and food security impacts of the 2017 El Niño flooding. Additionally, individuals with high social capital probably have better abilities to overcome the challenging socioeconomic post-disaster situation, and potentially progressively reinsert themselves into steady employment and ultimately improve their family’s food security and control PTSD stressors. Thus, identifying social capital characteristics, and trusted groups or community leaders could be an effective strategy to provide timely information, humanitarian resources and implementing health and societal initiatives to achieve both medium- and long-term disaster recovery.

### Limitations and strengths

Our findings should be interpreted at the light of key considerations. First, we did not study important additional mechanisms such as job security, resilience patterns, and family violence which likely influence long-term recovery outcomes as chronic PTSD, lack of individual and societal development, and malnutrition. Moreover, regarding the maternal-child dyad, it might be worth considering to study how parent roles in households, income levels, housing integrity may affect in PTSD, FS, and SC. Besides, other important children health outcomes could include studying impacts of natural events on access to pregnancy and pediatric care, and vaccination programs [53–56]. Second, we prioritized studying critically affected neighborhoods on the first-post disaster assessment, a potential sampling bias that could have overestimated the impact of El Niño assessed in our results. However, our estimates are probably valid because of the timely assessments and targeted questions addressing specifically outcomes resulting from the El Niño event [57]. Third, despite the participants’ risk of recall bias three to eight months after the event, their traumatic experiences and impacts in their lives potentially enhanced their recollections [58] and allowed us to identify frequent severe PTSD and food insecurity. Finally, we surveyed more participants in last two evaluations due to better accessibility and logistic conditions many months after the flooding, which led to disproportional groups to compare. However, we used valid post-estimation bootstrapping techniques to estimate difference between groups for the outcomes similarly conducted in other post-disaster studies [59].

Despite these caveats, our study has valuable strengths on study design and relevant findings for disaster planning and prevention. First, we studied a wide timeframe after the disaster, with assessments up to one year afterwards, presenting valuable field data to understand a complex public health issue. Moreover, our mixed method assessment clearly illustrates the challenging experiences confronted during disasters, which are hard to capture using traditional surveillance approaches. Finally, our conclusions likely remain valid despite time passed since the event. The main findings were drafted and locally discussed immediately after the field study concluded, with limited room for recollection biases. Also, the impact of future El Niño events will probably be similar, because their periodic nature and as social vulnerabilities of the historically affected communities remain unaddressed.

### Conclusions

PTSD and food insecurity were highly prevalent in the three months after El Niño, then consistently decreased until becoming virtually zero by one-year post-disaster. They not only have individual but also synergistic deleterious effects on affected families. High cognitive social capital may have mitigated the acute impact of the disaster, but most importantly it consistently increased for a year after the disaster and probably helped to counter long-term impacts and may build the resilience observed in the population.

## Supporting information

S1 Checklist Inclusivity in global research. (DOCX)

S1 Table Socioeconomic conditions of mothers affected by the 2017 El Niño flooding by cohort groups of enrollment in the Piura region, Peru, 2017–2018. (XLSX)

S1 Fig Critical affected areas due to high-rainfall and river floods in Piura, Castilla, and Catacaos—Peru, during the 2017 coastal El Niño. (A) The cities of Piura (west) and Castilla (east) divided by the Piura river. (B) The city of Catacaos (northward). Areas in red: basins without-mouth. Area in light blue: Piura river flood. Sources: Piura Center for Regional Emergency Operations, and The Copernicus Emergency Management Service–Mapping. Base layers of the Piura and Castilla districts are publicly available in Open Street maps (https://www.openstreetmap.org/#map=14/-5.1807/-80.6211), similarly for the Catacaos district (https://www.openstreetmap.org/#map=15/-5.2779/-80.6778), licensed under CC BY SA 2.0 (https://www.openstreetmap.org/copyright).
(TIFF)

S2 Fig Flowchart of participant selection among enrolled mothers from 2015–2018 cohort groups. (TIFF)
