# Supplementary figures and images for: Post-traumatic stress disorder, food insecurity, and social capital after the 2017 coastal El Niño flooding among mothers from Piura, Peru: A mixed method study
Source: PLOS Glob Public Health. 2024 Apr 18;4(4):e0002996. doi: 10.1371/journal.pgph.0002996 (PMC11025727; doi:10.1371/journal.pgph.0002996)

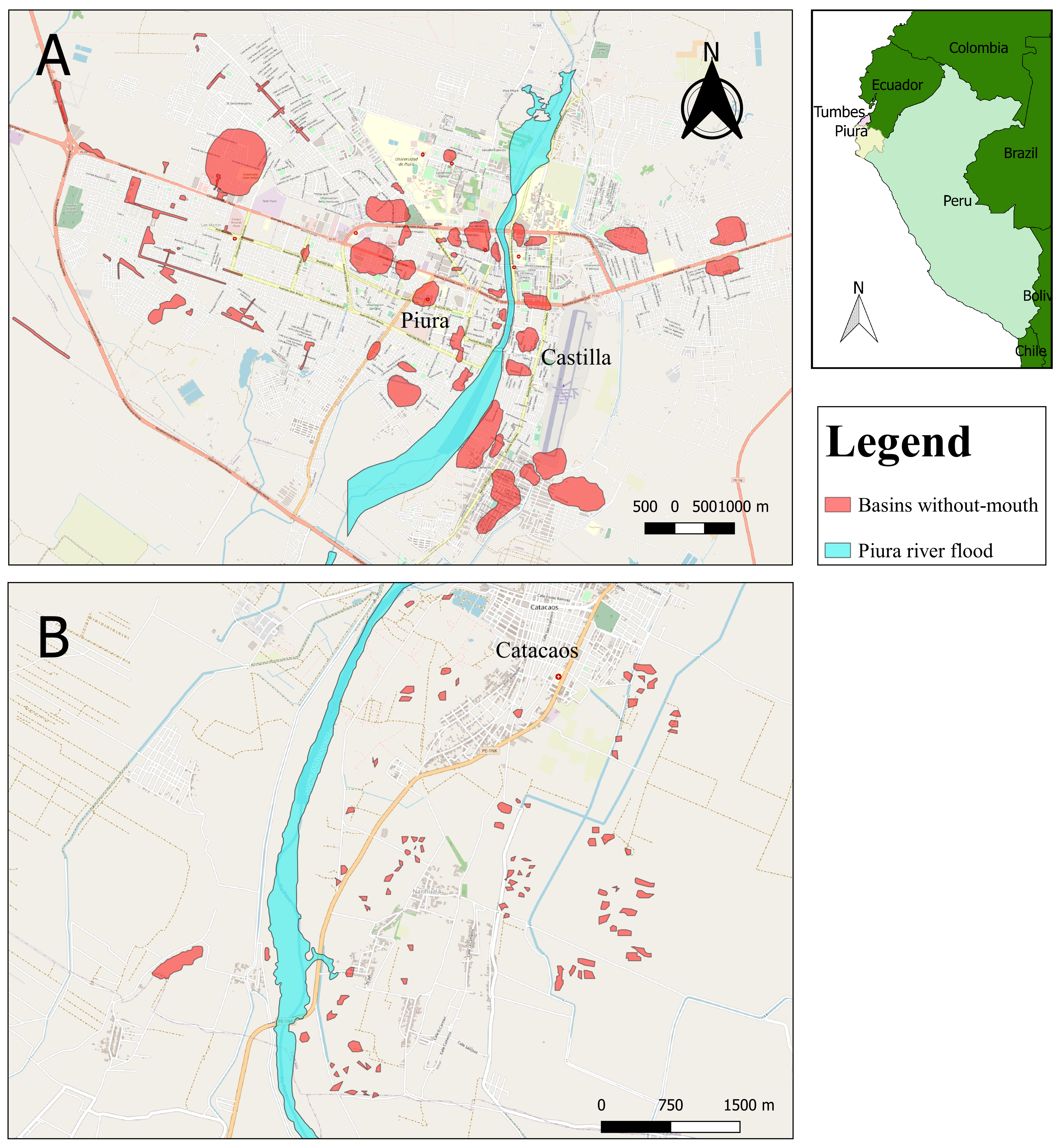

Supplement: S1 Fig — (A) The cities of Piura (west) and Castilla (east) divided by the Piura river. (B) The city of Catacaos (northward). Areas in red: basins without-mouth. Area in light blue: Piura river flood. Sources: Piura Center for Regional Emergency Operations, and The Copernicus Emergency Management Service–Mapping. Base layers of the Piura and Castilla districts are publicly available in Open Street maps (https://www.openstreetmap.org/#map=14/-5.1807/-80.6211), similarly for the Catacaos district (https://www.openstreetmap.org/#map=15/-5.2779/-80.6778), licensed under CC BY SA 2.0 (https://www.openstreetmap.org/copyright). (TIFF) [file pgph.0002996.s003.tiff]

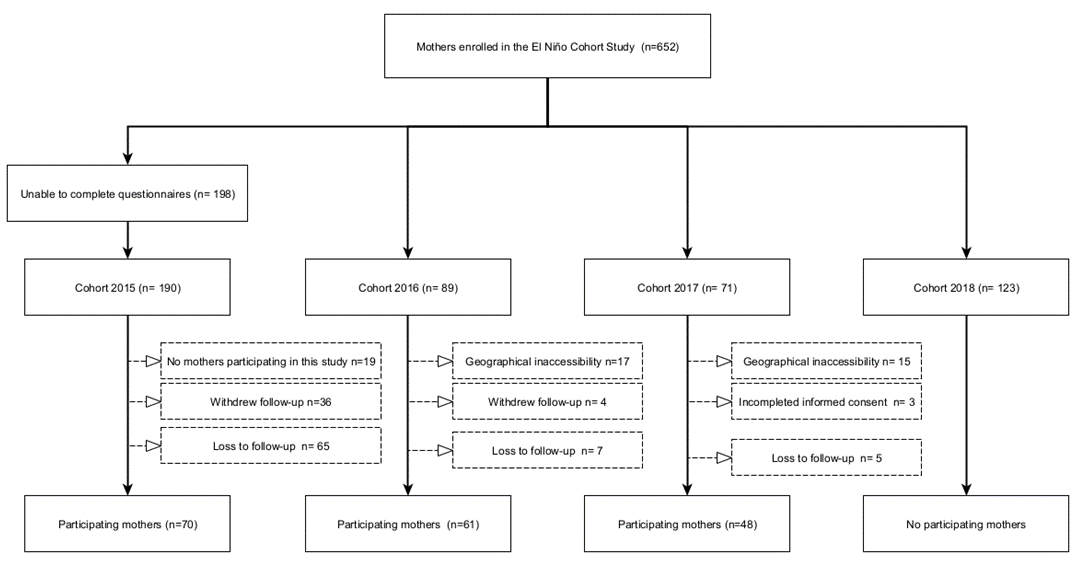

Supplement: S2 Fig — (TIFF) [file pgph.0002996.s004.tiff]
